# Early predictors and screening tool developing for severe patients with COVID-19

**DOI:** 10.1186/s12879-021-06662-y

**Published:** 2021-10-07

**Authors:** Le Fang, Huashan Xie, Lingyun Liu, Shijun Lu, Fangfang Lv, Jiancang Zhou, Yue Xu, Huiqing Ge, Min Yu, Limin Liu

**Affiliations:** 1 Zhejiang Provincial Center for Disease Control and Prevention, 3399 Binsheng Road, Binjiang District, Hangzhou, Zhejiang China; 2 Jingmen Center for Disease Control and Prevention, Jingmen, Hubei China; 3 Dongyang Center for Disease Control and Prevention, Dongyang, Jinhua China; 4 Sir Run Run Shaw Hospital, School of Medicine, Zhejiang University, Hangzhou, Zhejiang China

**Keywords:** Coronavirus disease 2019, Predictor, Screening, Severe patient

## Abstract

**Background:**

Coronavirus disease 2019 (COVID-19) is a declared global pandemic, causing a lot of death. How to quickly screen risk population for severe patients is essential for decreasing the mortality. Many of the predictors might not be available in all hospitals, so it is necessary to develop a simpler screening tool with predictors which can be easily obtained for wide wise.

**Methods:**

This retrospective study included all the 813 confirmed cases diagnosed with COVID-19 before March 2nd, 2020 in a city of Hubei Province in China. Data of the COVID-19 patients including clinical and epidemiological features were collected through Chinese Disease Control and Prevention Information System. Predictors were selected by logistic regression, and then categorized to four different level risk factors. A screening tool for severe patient with COVID-19 was developed and tested by ROC curve.

**Results:**

Seven early predictors for severe patients with COVID-19 were selected, including chronic kidney disease (*OR *14.7), age above 60 (*OR *5.6), lymphocyte count less than < 0.8 × 10^9^ per L (*OR *2.5), Neutrophil to Lymphocyte Ratio larger than 4.7 (*OR *2.2), high fever with temperature ≥ 38.5℃ (*OR *2.2), male (*OR *2.2), cardiovascular related diseases (*OR *2.0). The Area Under the ROC Curve of the screening tool developed by above seven predictors was 0.798 (95% *CI* 0.747–0.849), and its best cut-off value is > 4.5, with sensitivity 72.0% and specificity 75.3%.

**Conclusions:**

This newly developed screening tool can be a good choice for early prediction and alert for severe case especially in the condition of overload health service.

## Background

An unknown pneumonia emerged in Wuhan city, the capital of Hubei province in China, in December, 2019. A novel coronavirus was isolated by Chinese scientists from these patients with above unknown pneumonia in January, 2020, and this pneumonia was later designated coronavirus disease 2019 (COVID-19) in February, 2020, by World Health Organization (WHO) [1–3]. COVID-19 spread quickly in China and later became an international public health event. On March 11th, 2020, WHO declared COVID-19 as a pandemic. As of January 1st, 2021, there were more than 102 million cases with COVID-19 worldwide and more than 2 million of them died. The fatality rates of COVID-19 varied a lot in different countries: Yemen 29.00%, Mexico 8.50%, Egypt 5.60%, China 4.78%, Italy 3.47%, Australia 3.16%, The United Kingdom 2.78%, France 2.41%, United States 1.69%, Japan 1.46% [4]. There were many reasons for these fatality differences, including prevention strategy, health resource, proportion of elder population and others. However, one of the key measures to decrease the fatality rate is to strengthen early screening for severe patient with COVID-19 and timely medical treatment. Although there were several studies that had demonstrate various several predictors for severe patients with COVID-19, such as higher Sequential Organ Failure Assessment (SOFA), D-dimer greater than 1 μg/mL, decrease of CD8+ T cells, involvement of multiple lung lobes and pleural effusion. Some of the data of these predictors might not be available for all hospitals or all patients because of inadequate health facilities and professionals especially during a pandemic [5–7]. Even in the developed countries and high level hospitals, shortage of health resource was also a great challenge for them during COVID-19 pandemic [8]. What is more, some of these predictor were not early enough to prevent patients from becoming worse. It is very necessary to find early simple predictors for quick risk assessment to screen more potential severe patients with COVID-19 and give them timely treatment to decrease the number of severe patients and death [1,^ 8^–11].

## Methods

### Participants

This retrospective study included all the confirmed cases diagnosed with COVID-19 before March 2nd, 2020 in Jingmen city, a city of Hubei Province in China, which was 220 km far away from Wuhan city. Totally 813 cases were enrolled, excluding clinical diagnostic cases. These confirmed cases were divided into three groups including common cases, severe cases and fatal cases for characteristic comparison.

### Data collection

Data of patients with COVID-19 were collected through Chinese Disease Control and Prevention Information System which was the official disease reporting system for COVID-19 in China. All the data collection for COVID-19 control and prevention by local Center for Disease Control and Prevention and hospitals was legal. All the patients with COVID-19 had the obligation to cooperate with epidemiological survey according to ‘Law of the People's Republic of China on prevention and control of infectious diseases’. Each case had two parts of disease information. One was the Disease Report Card which included demographic information, hospital visit, disease onset time, diagnose time, death time, clinical category and outcome of patient with COVID-19, while another one was Epidemiological Survey Questionnaire which was consisted of exposure history, risk factors, symptoms, routine blood test and laboratory test results. The disease report card was fulfilled by doctors in hospitals, and epidemiological survey was conducted and inputted by health professionals in local county level Center for Disease and Prevention. All the disease information was examined and verified step by step from county level then by city level and finally by provincial level Center for Disease and Prevention. Disease information in Disease Report Card and Epidemiological Survey Questionnaire should be updated timely according to ‘Chinese COVID-19 surveillance programme’ in ‘Chinese prevention and control guideline for COVID-19’ [11].

### Definitions

All the definitions about COVID-19 were according to the Chinese management guideline for COVID-19 [12].

*Confirmed case* was defined as a suspected case with laboratory test COVID-19 positive from respiratory specimen by the Real-Time Reverse Transcription Polymerase Chain Reaction (RT-PCR) assay. For the confirmed case, it could be classified into four different clinical categories. 1. Mild type: with mild clinical symptoms or signs, without radiographic evidence of pneumonia. 2. Common type: having fever, respiratory and other symptoms, and with radiographic evidence of pneumonia. 3. Severe type: at least having one of following signs: (1) dyspnea, respiration frequency ≥ 30/ min, (2) finger oxygen saturation in resting condition ≤ 93%, (3) partial Pressure of arterial oxygen (PaO_2_) to Fraction of inspired oxygen (FiO_2_) ratio ≤ 300 mmHg, (4) radiographic evidence of lung infiltrates more than 50% within 24 to 48 h. 4. Critical type: at least having one of following conditions: (1) respiratory failure and in need of mechanical ventilation, (2) shock, (3) complication of other organ failure and in need of Intensive Care Unit (ICU) treatment. We defined 3 groups of confirmed patients in the data analysis: (1) common case, including survival cases of mild and common types, (2) severe case, including survival severe and critical types, (3) fatal case, referring to all the dead cases of above four types.

*Suspected case* was a case with at least one epidemiological exposure and at least two clinical signs, or a case with no explicit epidemiological exposure but at least three clinical signs as followings. 1. Epidemiological exposure during 14 days before disease onset: (1) travel to or living in Wuhan city or places around in Hubei province or other places with COVID-19 case, (2) exposure to people infected with COVID-19, (3) exposure to people with fever or respiratory symptoms from Wuhan city or places around in Hubei province or other places with COVID-19 case, (4) coming from family, workplace and school where occurred more than two COVID-19 cases. 2. Clinical signs: (1) having fever or respiratory symptoms, (2) radiographic evidence of COVID-19 pneumonia, (3) subnormal or normal white-cell count or subnormal lymphocyte count during early stage of disease onset.

### Statistical analysis

All statistical analysis was performed with SPSS software version 18.0, and *P* value less than 0.05 was considered statistically significant. The continuous variables were expressed as median (1st quartile, 3rd quartile) and were compared by Kruskal–Wallis test. And the categorical variables were presented as percentage and analyzed by Chi-square test. Multiple Logistic Regression by Hosmer and Lemeshow was used for developing the predictive model for screening severe patient with COVID-19, using forward stepwise approach with *P*_*(enter)*_ = 0.05 and *P*_*(remove)*_ = 0.10. The fit of the model was assessed by Nagelkerke R Square. All the predictors selected by the logistic regression were categorized to five different level risk factors according to their Odds Ratio (*OR*) values: (1) Not discernible: 0.9 ≤ *OR* < 1.1, (2) weak: 1.1 ≤ *OR* < 1.50, (3) moderate:1.50 ≤ *OR* < 3.0, (4) strong:3.0 ≤ *OR* < 7.0, (5) very strong: *OR* ≥ 7.0 [13]. For the not discernible, weak, middle, strong and very strong level risk factors, they were weighted as 0, 1, 2, 3 and 4 respectively. A screening tool for severe patient with COVID-19 was developed by these predictors and tested by Receiver Operating Characteristic (ROC) curve.

## Results

There were 37 fatal cases, 123 severe cases and 653 common cases (Table 1). Among these three different groups of COVID-19 patients, the age, gender and comorbidity were different. The median ages of fatal cases, severe cases and common cases were 61.0, 57.0 and 47.0 years old. The fatality rates in patients of age group 80–90, 70–79, 60–69, < 60, were 21.1% (4/19), 18.8% (9/48), 6.6% (9/137), 2.5% (15/609). The percentage of male in severe case group was 63.4%, higher than fatal case group (54.1%) and common case group (49.0%). Only 5.4% of fatal cases had no comorbidity, while the proportions of patient without comorbidity among severe cases and common cases was 59.1%, 77.8% respectively. The proportions of patients with two and three comorbidities in fatal cases was 21.6%, 8.1%, higher than the same proportion among severe case and common case groups. The exposures to Wuhan patients, diagnosed patients and symptomatic patients in the past 14 days, days from illness onset to visit hospital, days from visit hospital to be defined and the proportion of cluster case showed no statistical differences among the above three groups of patients with COVID-19.

**Table.1:** Characteristics of COVID-19 patients with different severity

Characteristics	Fatal cases (n = 37)	Severe cases (n = 123)	Common cases (n = 653)	χ^2^	*P*
Age	61.0 (55.0, 74.5)	57.0 (47.0, 68.0)	47.0 (35.0, 56.0)	78.7	< 0.001
Age groups				74.8	< 0.001
< 45	2.7%	20.3%	44.0%		
45–59	37.8%	34.1%	36.8%		
≥ 60	59.5%	45.6%	19.2%		
Male	54.1%	63.4%	49.0%	8.7	0.013
Exposure in the past 14 days					
Have been to Wuhan or other places with patient	60.0%	55.0%	51.2%	1.3	0.524
Exposure to diagnosed patient	30.6%	21.7%	30.4%	3.6	0.166
Exposure to symptomatic patient	33.3%	24.8%	33.9%	3.5	0.177
Days from illness onset to visit hospital	3.0 (1.0, 4.0)	3.0 (1.0, 5.0)	3.0 (1.0, 5.8)	0.1	0.953
Days from visit hospital to be defined	6.9 (5.2, 9.4)	7.5 (5.0, 9.9)	7.9 (5.4, 10.9)	0.9	0.653
Cluster case	24.1%	19.6%	28.5%	3.5	0.177
Comorbidity				103.4	< 0.001
None	5.4%	59.1%	77.8%		
One disease	64.9%	26.1%	15.0%		
Two diseases	21.6%	13.0%	6.0%		
Three diseases and more	8.1%	1.8%	1.2%		

Apart from chronic lung disease and liver disease, the proportions of underlying comorbidities such as hypertension, diabetes, cardiovascular disease, chronic kidney disease and other diseases were significantly different among COVID-19 patients with different severity, see Table 2. In the fatal cases, 59.5% cases were with hypertension, 21.6% with diabetes, 21.6% with cardiovascular diseases and 16.2% with chronic kidney disease, higher than the same prevalence rates of the same comorbidity among severe cases and common cases.

**Table.2:** Comorbidities among COVID-19 patients with different severity

Comorbidity	Fatal cases (n = 37)	Severe cases (n = 123)	Common cases (n = 653)	χ^2^	*P*
Hypertension	59.5%	17.4%	12.6%	58.7	< 0.001
Diabetes	21.6%	7.0%	3.9%	22.8	< 0.001
Cardiovascular diseases	21.6%	5.2%	4.1%	22.2	< 0.001
Chronic kidney disease^a^	16.2%	7.0%	0.3%	58.6	< 0.001
Chronic lung disease^a^	2.7%	2.6%	2.0%	0.3	0.874
Chronic liver disease^a^	0	1.7%	0.7%	1.8	0.412
Other diseases	13.5%	16.5%	7.0%	12.0	0.002

^a^Chronic referred to the disease lasting more than 3 months

There were more than 18 symptoms among COVID-19 patients (Table 3). The most prevalent symptoms in common cases were fever (81.2%), cough (36.3%), sputum (23%) and fatigue (18.5%). These symptoms were also very common in severe and fatal cases. Besides above symptoms, headache (16.7%), vomiting (13.9%), nausea (11.1%) and myalgia (13.9%) were another frequently reported symptoms in fatal cases. The proportions of high fever (temperature ≥ 38.5 ℃), vomiting, nausea were significantly different among these three groups of patients.

**Table 3:** Symptoms of COVID-19 patients with different severity

Symptoms	Fatal cases (n = 37)	Severe cases (n = 123)	Common cases (n = 653)	χ^2^	*P*
Fever (temperature > 37.3℃)	94.4%	84.3%	81.2%	4.5	0.107
High Fever (temperature ≥ 38.5℃)	47.1%	43.2%	31.4%	7.7	**0.022**
Cough	30.6%	45.2%	36.3%	4.1	0.132
Sputum	25.0%	18.3%	23.0%	1.4	0.492
Fatigue	25.0%	20.9%	18.5%	1.2	0.548
Headache	16.7%	9.6%	8.0%	3.4	0.185
Vomiting	13.9%	6.1%	2.6%	14.5	**0.001**
Nausea	11.1%	7.0%	3.3%	7.8	**0.020**
Myalgia	13.9%	13.9%	9.5%	2.6	0.278
Arthralgia	8.3%	3.5%	3.4%	2.4	0.313
Breathlessness	8.3%	3.5%	4.1%	1.7	0.427
Dyspnea	5.6%	3.5%	2.6%	1.2	0.543
Chest distress	8.3%	5.2%	9.2%	1.9	0.382
Chest pain	0	0.9%	0.8%	0.3	0.859
Nasal obstruction	0	0.9%	3.8%	3.9	0.144
Runny nose	2.8%	5.2%	4.9%	0.4	0.830
Diarrhoea	2.8%	9.6%	7.7%	1.8	0.410
Stomach ache	0	0	1.0%	1.5	0.474
Conjunctival congestion	0	0	0.2%	0.3	0.884

White blood count showed no significant difference among fatal, severe and common cases, and 55.7% to 69.2% patients had normal level white blood cell count (Table 4). However, lymphocyte count, lymphocyte constituent ratio, neutrophil granulocyte constituent ratio, Neutrophil to Lymphocyte Ratios (NLR) and the proportion of NLR > 4.7 were different among above three groups of COVID-19 patients. There were 78.3% of fatal cases and 68.4% of severe cases with subnormal lymphocyte count, while only 46.1% patients had subnormal (including significant decreased) lymphocyte count in common cases. The lymphocyte constituent ratios of fatal, severe, and common cases were 18.7%, 21.3% and 26.0%, while neutrophil granulocyte constituent ratios of these three groups of COVID-19 patients were 75.4%, 67.3% and 62.1% respectively. The Neutrophil to Lymphocyte Ratios (NLR) in fatal and severe case groups were 4.2 and 3.1, larger than the NLR (2.4) in common case group. The proportions of NLR > 4.7 among fatal, severe and common cases were 44.4%, 34.7% and 15.0%.

**Table 4:** White blood cell of COVID-19 patients with different severity

	Fatal cases (n = 37)	Severe cases (n = 123)	Common cases (n = 653)	χ^2^	*P*
**White blood cell count, × 10**^**9**^ **per L**	5.5 (4.0,7.2)	4.7 (3.4,6.2)	4.6 (3.7,5.8)	3.7	0.154
Subnormal (< 4)	23.1%	36.1%	35.1%	8.0	0.094
Normal (4–10)	69.2%	55.7%	61.9%		
Above normal (> 10)	7.7%	8.2%	3.1%		
**Lymphocyte count, × 10**^**9**^ **per L**	0.8 (0.6,1.1)	0.9 (0.6,1.3)	1.1 (0.9,1.5)	27.3	< 0.001
Significant decreased (< 0.8)	43.5%	49.5%	21.1%	43.9	< 0.001
Subnormal (0.8–1.09)	34.8%	18.9%	25.0%		
Normal (1.1–3.2)	17.4%	28.4%	52.2%		
Above normal (> 3.2)	4.3%	3.2%	1.7%		
**Lymphocyte constituent ratio (%)**	18.7 (11.6,23.4)	21.3 (11.8,28.6)	26.0 (18.6,32.7)	25.3	< 0.001
Subnormal (< 20%)	59.3%	46.4%	29.9%	19.8	0.001
Normal (20%-40%)	37.0%	49.5%	59.7%		
Above normal (> 40%)	3.7%	4.1%	10.4%		
**Neutrophil granulocyte constituent ratio (%)**	75.4 (60.3,81.3)	67.3 (56.7,76.8)	62.1 (54.3,70.5)	19.9	< 0.001
Subnormal (< 20%)	3.7%	11.3%	6.8%	41.6	< 0.001
Normal (40%-75%)	44.4%	60.8%	80.4%		
Above normal (> 75%)	51.9%	27.8%	12.8%		
**Neutrophil-to-Lymphocyte Ratio (NLR)**	4.2 (2.6, 7.4)	3.1 (2.0, 6.2)	2.4 (1.7,3.8)	22.0	< 0.001
Increased NLR (> 4.7)	44.4%	34.7%	15.0%	30.7	< 0.001

The variables showed statistical significance in the Tables 1, 2, 3 and 4, such as age group (age above 60), gender (male proportion), chronic kidney disease, cardiovascular related diseases, other diseases, higher fever, vomiting, nausea, lymphocyte count group, Neutrophil to Lymphocyte Ratio group were included in the following logistical regression analysis. It showed that chronic kidney disease and age above 60 were very important indicators for severe patients with COVID-19, and their *OR* values were 14.7 and 5.6 respectively (Table 5). Other risk factors such as lymphocyte count less than < 0.8 × 10^9^ per L (*OR *2.5), NLR larger than 4.7 (*OR *2.2), high fever with temperature ≥ 38.5℃ (*OR *2.2), male (*OR *2.2), cardiovascular related diseases (*OR *2.0) were also good predictors for severe patients.

**Table 5:** Early predictors for severe patients with COVID-19

	*P*	*OR* (95% *CI*)
Age above 60	< 0.001	5.6 (3.1, 9.9)
Gender (male)	0.005	2.2 (1.3, 3.7)
Chronic kidney disease	0.026	14.7 (1.4, 157.1)
Cardiovascular related diseases^a^	0.030	2.0 (1.1, 3.7)
High Fever (temperature ≥ 38.5 ℃)	0.005	2.2 (1.3, 3.8)
Significant decreased lymphocyte count (< 0.8 × 10^9^ per L)	0.002	2.5 (1.4, 4.5)
Increased NLR (> 4.7)	0.012	2.2 (1.3, 3.8)

Multiple logistic regression conducted in this analysis. Due to the small sample size of fatal cases, severe patients with COVID-19 in this model included both severe cases and fatal cases. Common case was used as reference group

^a^Cardiovascular related diseases included hypertension, diabetes and cardiovascular diseases in Table 2

According to the *OR* values of above predictors, all the selected predictors were categorized into different level risk factors (Table 6): (1) very strong risk factor: chronic kidney disease, weighted as 4, (2) strong risk factor: age above 60, weighted as 3; (3) moderate risk factors: male, with at least one cardiovascular related disease, high fever (temperature ≥ 38.5℃), lymphocyte count < 0.8 × 10^9^ per L, NLR > 4.7, all weighted as 2. The risk of a COVID-19 patient for becoming a severe patient was measured by this screening tool, and its risk score equaled to the total score of these seven predictors in this screening tool.

**Table 6:** Screening tool for severe patients with COVID-19

Predictor	Score^a^	
	No	Yes
Age above 60	0	3
Male	0	2
With chronic kidney disease	0	4
With at least one cardiovascular related disease (hypertension, diabetes, stroke, heart disease)	0	2
High Fever (temperature ≥ 38.5 ℃)	0	2
Lymphocyte count < 0.8 × 10^9^ per L	0	2
Neutrophil-to-lymphocyte ratio > 4.7	0	2

The risk of a COVID-19 patient for becoming a severe patient can be measured by this screening tool, and its risk score equaled to the total score of above seven predictors

^a^According to the OR values of above predictors, they were categorized into different level risk factor: not discernible, weak, middle, strong and very strong level risk factors, their risks were weighted as 0, 1, 2, 3 and 4 respectively

**Fig. 1:**
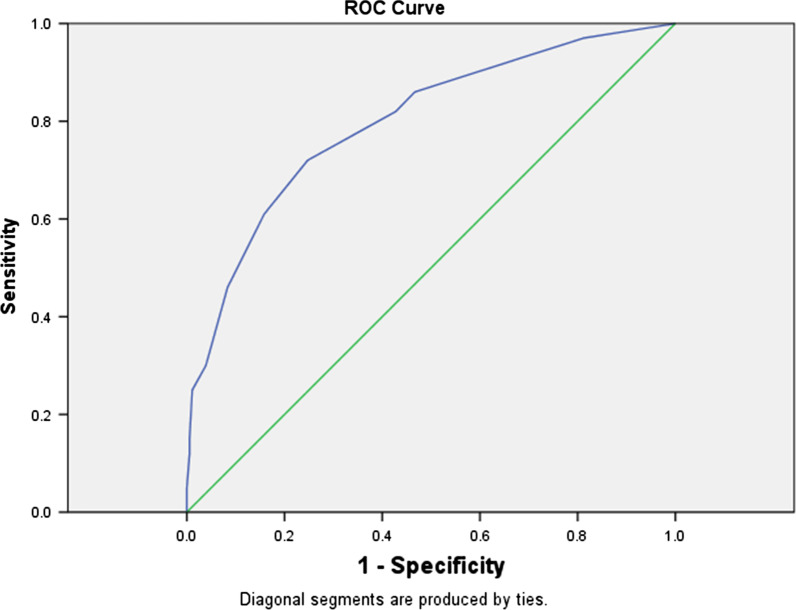
The ROC curve for the screening tool for severe patients with COVID-19

The Area Under the ROC Curve (AUC) was 0.798 (95% Confidence Internal (*CI*) 0.747–0.849), and its best cut-off value was > 4.5, with sensitivity 72.0% and specificity 75.3% (Fig. 1).

## Discussions

COVID-19 is a rapidly progressive disease. Zhou et al. found that the non-survivor of COVID-19 usually developed more complications by day 15, and died by day 19 [5]. So it is crucial to screen risk population for severe patients with COVID-19 as early as possible. During a pandemic or in an inadequate health resource scenario, it is unfeasible to do lots of expensive or time–cost laboratory tests and physical examination for all patients [9,^ 14^, 15]. Therefore, it is very important to find quick and low-cost screening tool to predict the possibility of becoming severe patients with COVID-19.

Similar to most other previous reports, patients with conditions including elder age (usually elder than 60 years old), male, underlying chronic diseases including cardiovascular disease, diabetes, kidney disease were more likely to suffer from severe COVID-19 infection and death [3,^ 5^,^ 9^,^ 14^,^ 16^–18]. What needs to be stressed is that elder age is still an independent risk factor for severe patient after considering the confounding effects of underlying chronic diseases. This information should be emphasized among elder population without underlying medical condition, because they usually have overconfidence of their health and weak awareness of self-protection. Kidney disease demonstrated more risk than other chronic diseases and the *OR* value reached 14.7. The mechanism of kidney disease involvement in patients with COVID-19 is likely to be multidimensional. First, the novel coronavirus may exert direct cytopathic effects on kidney tissue and worsen its organ function. Second, deposition of immune complexes of viral antigen or virus-induced specific immunological effector mechanisms (specific T-cell lymphocyte or antibody) may further damage the kidney. Third, virus-induced cytokines or mediators might exert indirect effects on renal tissue, such as hypoxia, shock, and rhabdomyolysis [7,^ 19^, 20]. Early detection and effective intervention of kidney disease involvement may help to reduce deaths of patients with COVID-19.

Symptoms and signs are another set of valuable health data that may be available for all patients. The most common onset symptoms of COVID-19 onset are fever, cough, sputum, fatigue, headache, vomiting, nausea, et al., and most of them are not specific [3,^ 9^,^ 18^,^ 21^, 22]. However, high fever was proved to be a good predictor for severe cases. This finding is extremely important for guiding the public to self-judge whether he/she needs to go to hospital for treatment. In addition, routine blood test is quite prevalent in all level hospitals even in developing countries, and it is another good choice for quick risk evaluation. Along with most other studies, this research showed that lymphocyte count less than < 0.8 × 10^9^ per L, NLR larger than 4.7 were early predictors for severe patients with COVID-19. The decrease of lymphocyte which finally leads to immune disorder is due to the sustained responses of cytokines and chemokines (namely cytokine storm) caused by novel coronavirus, due to the relative distribution of ACE1 vs ACE2 receptors in the surrounding epithelium [23–26]. The NLR is an important supplement for lymphocyte count, reflecting the inflammation of the patient which indicates the possibility of bacteria infection [8, 27].

The most frequently reported predictors for severe patients with COVID-19 in other researches included age, comorbidities, vital signs, image features, sex, lymphocyte count, and C reactive protein [28]. The screening tool developed by this study included six of above predictors, except image features and C reactive protein. Image features and C reactive protein test were not as prevalent as routine blood test (such as lymphocyte count) in low-level medical center. What is more, most studies did not transfer the prediction model into risk scoring scale. So this newly developed screening tool is more useful than other prediction models.

The strengths of this study were: (1) The predictors were easily accessed and the screening could be broadly used. (2) The representativeness of COVID-19 in this study was good. It included all the patients with COVID-19 in the whole city while most other studies only recruited COVID-19 cases based on hospitals. The limitations were just as followings: (1) Laboratory tests were limited and not dynamic, so the predictors may not be comprehensive. (2) The predictors for severe case and death were not stratified for multifactor analyses due to the small amount of death, and the differences between sever case and death were not further explored. (3) The sample size of this study was relatively small, and a different mix of comorbidities in another population of equal size might find different odds ratios. So the generalization of this newly developed risk screening tool needs more test among other populations.

## Conclusions

The screening tool by seven indicators including chronic kidney disease, age, lymphocyte count, Neutrophil to Lymphocyte Ratio, high fever, male and cardiovascular related diseases, can be used for early prediction of severe patients with COVID-19. All the information required for prediction can be potentially obtained from quick epidemiological inquiry and routine blood test. It can help screen for more potential risk patient by limited health resource and offer timely treatment to save more patients. It is very cost-effective and deserves widely applications under the condition of overload health service.

## Data Availability

The datasets used and/or analysed during the current study are not publicly available due to the IRB policy, however are available from the corresponding author on reasonable request.

## Abbreviations

COVID-19: Coronavirus disease 2019
WHO: World Health Organization
SOFA: Sequential Organ Failure Assessment
RT-PCR: Real-Time Reverse Transcription Polymerase Chain Reaction
PaO2: Pressure of arterial oxygen
FiO2: Fraction of inspired oxygen
ICU: Intensive Care Unit
OR: Odds ratio
ROC: Receiver operating characteristic
AUC: Area under the curve
CI: Confidence internal
NLR: Neutrophil to lymphocyte ratios
ARDS: Acute respiratory distress syndrome
